# *Brassica oleracea* var *italica* and Their By-Products as Source of Bioactive Compounds and Food Applications in Bakery Products

**DOI:** 10.3390/foods13213513

**Published:** 2024-11-02

**Authors:** Jhazmin Quizhpe, Pablo Ayuso, María de los Ángeles Rosell, Rocío Peñalver, Gema Nieto

**Affiliations:** Department of Food Technology, Nutrition and Food Science, Veterinary Faculty, University of Murcia, Regional Campus of International Excellence “Campus Mare Nostrum”, Campus de Espinardo, 30100 Murcia, Spain; jhazminedith.quizhper@um.es (J.Q.); pablo.ayuson@um.es (P.A.); mariaangeles.rosellp@um.es (M.d.l.Á.R.); rocio.penalver@um.es (R.P.)

**Keywords:** broccoli, glucosinolates, chemoprevention, green extraction techniques, functional food

## Abstract

Broccoli (*Brassica oleracea* var. *italica*) is one of the most consumed cruciferous crops in the world, with China and Spain acting as the main producers from outside and within the EU, respectively. Broccoli florets are edible, while the leaves and stalks, discarded in the field and during processing, are by-products. Therefore, the objective of this study was to conduct a comprehensive review of the nutrient and phytochemical composition of broccoli and its by-products, as well as its beneficial effects. In addition, the study highlights the revalorization of broccoli by-products through innovative green technologies and explores their potential use in bakery products for the development of functional foods. The studies suggested that broccoli is characterized by a high content of nutrients and bioactive compounds, including vitamins, fiber, glucosinolates, and phenolic compounds, and their content varied with various parts. This high content of value-added compounds gives broccoli and its various parts beneficial properties, including anti-cancer, anti-inflammatory, antioxidant, antimicrobial, metabolic disorder regulatory, and neuroprotective effects. Furthermore, broccoli and its by-products can play a key role in food applications by improving the nutritional profile of products due to their rich content of bioactive compounds. As a result, it is essential to harness the potential of the broccoli and its by-products that are generated during its processing through an appropriate agro-industrial revalorization, using environmentally friendly techniques.

## 1. Introduction

Broccoli (*Brassica oleracea* var. *italica*) is a biennial crop belonging to the *Brassicaceae* family, genus *Brassica*, along with cabbage, cauliflower, kale, turnip, mustard, and Brussels sprouts [1]. The *Brassicaceae* family contains up to 338 genera and 3709 species of angiosperm dicotyledonous plants characterized by a Greek cross structure of the four petals that form the flower distributed all around the globe in all continents excluding Antarctica [2,3]. The term “broccoli” comes from the Italian plural of “broccolo”, which means “the flowering crest of a cabbage” [4]. As for its origin, the leading hypothesis is that broccoli evolved from wild ancestors in the eastern part of the Mediterranean [5,6] and was subsequently introduced in the United Kingdom in the 1700s and in the United States and China in the 1800s [7].

**Figure 1:**
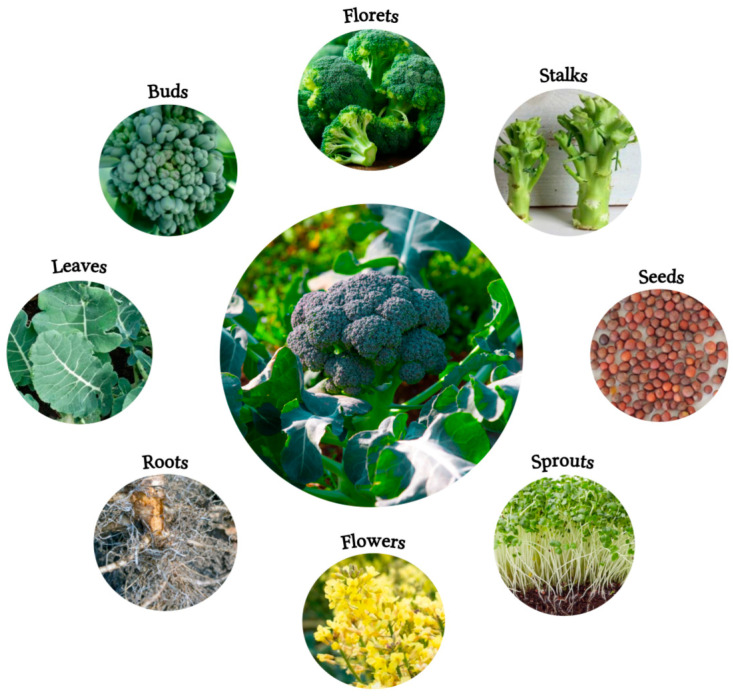
Botanical parts of broccoli (*Brassica oleracea* var *italica*).

Broccoli is an important crop worldwide, as its production, along with cauliflower, reached approximately 26.06 million tons globally in 2022, a significant increase from 12.20 million tons in 1994 [8]. The largest broccoli producers in 2022 were mainland China and India in Asia (81%), Spain and Italy in Europe (8.3%), and the United States and Mexico in the Americas (8.2%) [8]. Within Spain, more than 45% of the total broccoli production comes from the Region of Murcia, with a total area of 13,340 ha and a production of 18,830 kg/ha [9]. Broccoli crops have numerous botanical parts (Figure 1) depending on their stage of maturity. The fresh weight (FW) of a mature broccoli can reach 776 g, where only the florets are the edible part (representing only up to 15% of the plant), while the leaves, stems, and roots (47, 21, and 17% of the total weight) are discarded at harvest, considered as by-products [10,11].

The beneficial effects of vegetables and their by-products have been widely studied in recent years as a source of valuable bioactive compounds (BACs) with potential application in the treatment and prevention of human diseases (diabetes, cancer, inflammation, lipid oxidation, cardiovascular diseases (CVD), obesity, and urinary tract infection, among others) [12]. The high content of (BACs) in broccoli and its by-products, such as glucosinolates (GSLs), phenolic compounds (PCs), carotenoids, vitamin C, dietary fiber (DF), and minerals, provide beneficial effects against certain diseases, such as cardiometabolic diseases and cancer [13]. Numerous studies have determined that broccoli has anticarcinogenic, anti-inflammatory, antimicrobial, antioxidant, antidiabetic, and antihypertensive effects [14,15,16,17,18]. For example, in a study conducted in 2019, peptides were purified and identified from broccoli hydrolysates and evaluated for their angiotensin-converting enzyme inhibitory activity in rats, where they demonstrated a significant hypotensive effect in vivo [15].

In recent decades, the European Commission has adopted a series of proposals with the aim of reducing net greenhouse gas emissions by 2030 [19]. In the European Union, up to 20% of the food produced is wasted, especially fruit and vegetables, leading to significant environmental and economic challenges [20,21]. Due to these problems, the revalorization of by-products using “green technologies” is of great interest, since it works with safe and environmentally friendly solvents, while at the same time promoting the circular economy [22,23]. Furthermore, a principal objective in the valorization of by-products is their incorporation into food products with the dual purpose of preserving or enhancing nutritional value and meeting the growing consumer demand for safer and healthier options [24,25,26].

This literature review synthesizes and analyzes the nutritional composition and BACs of the various parts of broccoli, as well as the latest advances in their beneficial health effects and novel and green compound extraction techniques. Furthermore, it addresses an innovative aspect: the revalorization of *Brassica oleracea* var. *italica* and its by-products for the enrichment of bakery products. This approach allows for a more optimal use and exploitation of the health benefits of broccoli, also responding to the growing demands of sustainability and the development of functional foods, contributing to the advancement in the production of healthier and environmentally friendly products.

## 2. Materials and Methods

To conduct this comprehensive review, many articles were searched in the databases Science Direct, Google Scholar, PubMed, Web of Science, Scopus, and Dialnet. The final search was carried out in August 2024 and included reviews and research, reports, and theses in both English and Spanish. The keyword “broccoli” was used in combination with other terms such as “glucosinolates”, “phenolic acids”, “antioxidant”, “anticancer”, “anti-inflammatory”, “bakery products”, “extraction techniques”, “food waste”, or “by-products”. Once the search was completed, the abstracts and specific sections of the articles were read to check whether the studies analyzed the different botanical parts of broccoli in at least one of the four dimensions on which this review focuses (nutritional properties, health benefits, extraction techniques of BACs, and food application). Studies that met these criteria were then summarized and synthesized for inclusion in this comprehensive review.

## 3. Nutritional Composition

### 3.1. Macronutrients

The different botanical parts of broccoli, which include florets, stems, leaves, and sprouts, present important variations in their nutritional composition (Table 1). The condition of the sample (fresh or dry) and the genetic, developmental, geographical, and environmental factors may also be responsible for these variations.

The different botanical parts of broccoli provide a range of 24–51 kcal per 100 g of sample, being lower in the sprouts and higher in the leaves [1,27,28]. Moreover, in terms of moisture, all parts contain a high amount of water (85 to 91%) [29,30]. Broccoli is considered a low-calorie food due to these low levels of energy value combined with its high water content.

#### 3.1.1. Proteins

Broccoli is a major source of vegetal proteins in all its botanical parts. According to Dufoo-Hurtado et al. [31] and López-Cervantes et al. [33] proteins represent up to 31.28, 18.14, 27.49, and 43.16 g per 100 g dry weight (DW) of florets, stalks, leaves, and sprouts, respectively. In terms of FW, this content is significantly lower, with the highest level reported for the sprouts (up to 3 g/100 g FW) [28]. The amino acids (AAs) profile also varies for the different botanical parts. In general, florets, stalks, and leaves have a remarkably similar profile, with tyrosine being the most abundant with 70, 32, and 35 mg/g DW, respectively. Their essential amino acids (EAAs) content is approximately 23% in florets and stalks and 26% in leaves [31]. On the other hand, sprouts differ from this profile, where glutamic acid is the most predominant with levels up to 144 mg/g DW. In addition, their percentage of EAAs varies according to the days of germination, ranging from 34% (seeds) to 37% (3 days of germination) and then to 26% (11 days of germination) (Table 1).

#### 3.1.2. Sugars

In broccoli sprouts, the carbohydrate content decreases with germination, being higher in seeds than in 11-day-old sprouts. According to Li et al. [37], this decrease could be related to starch hydrolysis during the germination process, a component that is not detected in mature broccoli. Total sugar content is higher in leaves (25.39 g/100 g DW) and florets (24.61 g/100 g DW) than in stalks (18.58). Moreover, reducing sugars (RS) constitute an important part of this group, reporting ranges from 2.72 (stalks) to 7.07 (leaves) g/100 g DW [35]. In general, the most predominant sugars in broccoli are uronic acid, glucose, arabinose, fructose, and galactose [38,39]. According to Femenia et al. [40], this sugar profile is correlated with the presence of pectin polysaccharides and cellulose as main components of *Brassicaceae* family cell walls.

#### 3.1.3. Dietary Fiber

DF consists of a mixture of carbohydrates and polymers present in plants, such as cellulose, hemicellulose, peptide substances, gums, resistant starch, and inulin [41]. Furthermore, DF is composed of two fractions: a soluble (SDF) one that can form viscous gels in contact with water in the intestinal tract and an insoluble (IDF) one that does not form gels but can retain water in its structural matrix, increasing intestinal transit [42]. DF content in broccoli also depends on the plant section, being lower in the sprouts (2.1 g/100 g DW) and higher in the stalk (77.28 g/100 g DW). The edible part, the florets, contain around 64 g/100 g DW, which is proportionally higher than in other species of the *Brassicaceae* family such as Brussels sprouts, cauliflower, or turnips [43]. On the other hand, all parts of broccoli contain more IDF (up to 66.18 g/100 g DW) than SDF (up to 11.10 g/100 g DW) (Table 1). In general, the stalk is the part with the highest amount of insoluble fiber, which is due to the physiological functions of the stalks; most of the polymers in their walls are not soluble in water [43]. In addition, the amount of DF can be influenced by certain factors, such as storage conditions or type of field [44,45].

#### 3.1.4. Fat Content

The fat content of broccoli is 4.59, 6.58, and 6.72 g/100 g DW in florets, stalks, and leaves, respectively [29]. For sprouts, their lipid content was found to decrease during germination, dropping from 8.67 to 3.16 g/100 g DW after 11 days of germination [33]. Moreover, all these parts are characterized by a profile rich in polyunsaturated fatty acids (PUFAs), representing 55–71% of the total fatty acids (FAs). The most abundant FAs in the edible part are α-linolenic acid with 43%, followed by palmitic acid and linoleic acid with 22% and 20%, respectively. Finally, broccoli also displays a good n-6/n-3 ratio of 1.5, being in accordance with the established recommendations (ranges of 1–5/1) [46], which is also lower than in other plants such as cabbage (2.1–2.6), tomato (41–46), or carrot (26–27) [47].

### 3.2. Micronutrients

Broccoli and its by-products represent a valuable source of important minerals that contribute to overall health and well-being [48]. Compared to the mineral composition of other vegetables reported in the literature, the levels of potassium (K), calcium (Ca), iron (Fe), and zinc (Zn) are elevated in broccoli (Table 2). Deficiency of these minerals can lead to different disorders such as anemia [49], osteoporosis [50], CVD [51], renal, and immunological disorders [52]. The macro- and micro-element profile varies depending on the botanical part, but, in general, the florets, stalks, and leaves have several similarities between them and differ from the sprouts. The cruciferous vegetables (*Brassicaceae*) are characterized by their ability to accumulate the essential micro-element selenium and synthesize seleno-compounds, which have beneficial health effects, such as cancer-preventive properties [53]. Broccoli, through selenium salts, synthesizes and accumulates selenium-methylselenocysteine, being the predominant form of selenium in this vegetable [54].

Broccoli also has a high content of vitamins A, B9, C, E, and K (Table 2). These vitamins have been characterized in florets, presenting some lack of knowledge in leaves but especially in stalks and sprouts. Despite this, Liu et al. [10] have determined that leaves have much higher levels of vitamin E and K than florets. In addition, it has been demonstrated that the content of vitamin C in broccoli is reduced after cooking methods, such as conventional cooking, steaming, and sous-vide, with a loss of 62, 42, and 37%, respectively [55]. Therefore, broccoli products with the consideration of this vitamin as a quality attribute should be minimally processed.

**Table 2:** Micronutrients of diverse botanical parts of broccoli.

Composition	Unit	Florets	Stalks	Leaves	Sprouts	Ref.
Minerals						
Macro-element						
Calcium	(mg/g DW)	4.65	7.10	28.99	-	[10]
	(mg/g FW)	-	-	-	0.88	[56]
Magnesium	(mg/g DW)	1.78	1.67	1.33	-	[10]
	(mg/g FW)	-	-	-	0.51	[56]
Phosphorus	(mg/g DW)	7.01	5.07	3.42	-	[10]
	(mg/g FW)	-	-	-	0.69	[56]
Potassium	(mg/g DW)	145.00	182.00	136.00	-	[10]
	(mg/g FW)	-	-	-	3.26	[56]
Sodium	(mg/g DW)	0.39	6.43	2.63	-	[10]
	(mg/g FW)	-	-	-	0.52	[56]
Micro-element						
Copper	(μg/g DW)	0.29	0.24	0.21	-	[10]
	(μg/g FW)	-	-	-	0.9	[56]
Iron	(μg/g DW)	45.83	15.83	40.50	-	[10]
	(μg/g FW)	-	-	-	6.70	[56]
Manganese	(μg/g DW)	18.83	7.00	26.17	-	[10]
	(μg/g FW)	-	-	-	3.70	[56]
Selenium	(μg/100 g FW)	2.50	-	-	-	[57]
Zinc	(μg/g DW)	54.00	22.67	23.33	-	[10]
	(μg/g FW)	-	-	-	3.70	[56]
Vitamins						
Vitamin A	(μg/g FW)	0.08	-	-	-	[58]
Vitamin B9	(μg/g FW)	0.65	-	-	-	[58]
Vitamin C	(mg/g DW)	2.54	-	2.92	0.51	[59,60]
	(mg/g FW)	0.91	-	-	12.41	[30,58]
Vitamin E	(μg/g DW)	1.57	1.97	155.00	-	[10]
	(μg/g FW)	1.50	-	-	-	[58]
Vitamin K	(μg/g DW)	8.84	2.21	24.30	-	[10]
	(μg/g FW)	1.02	-	-	-	[58]

FW: fresh weight; DW: dry weight.

### 3.3. Bioactive Compounds

Numerous BACs, including organosulfur compounds, carotenoids, and PCs, have been identified from the various parts of broccoli, especially from florets, stalks, leaves, and sprouts. However, a large variety of these compounds show values that varied from low to elevated concentrations depending on the botanical part, as well as on external factors such as growing media, harvest, postharvest, or even extraction procedures. Table 3 summarizes these BACs.

#### 3.3.1. Organosulfur Compounds

GSLs, sulfur compounds responsible for the characteristic aroma and flavor of broccoli, represent one of the most important groups of broccoli constituents. Biological activities such as fungicidal, bactericidal, nematocidal, anti-cancer, antioxidant, and anti-inflammatory have been attributed to these compounds and their different breakdown products [17,61,62].

**Figure 2:**
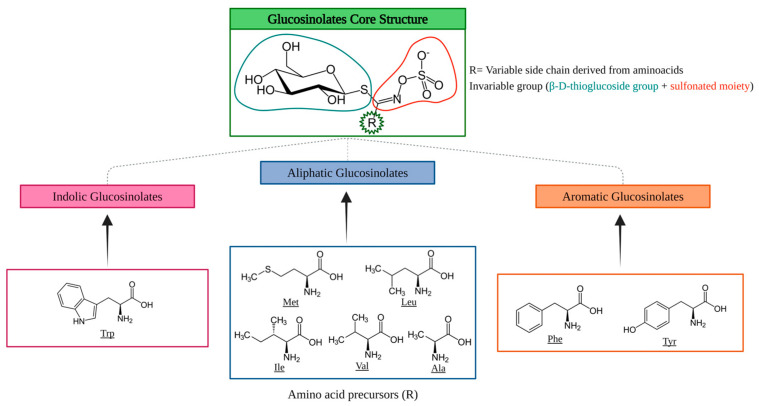
Basis structure and classification of GSLs according to the type of precursor AAs.

GSLs are water-soluble secondary plant metabolites whose structure consists of an invariable chain composed of a sulfonated moiety linked to a β-D-thioglucose group and a variable chain derived from different AAs [63]. Based on their precursor AAs, GSLs can be classified as aliphatic, aromatic, and indolic (Figure 2).

In general, these compounds are chemically and thermally stable until they are hydrolyzed by the enzyme myrosinase (EC 3.2.1.147, also known as synigrase or thioglucosidase). This is an S-glucosidase enzyme that catalyzes the hydrolysis of thioglucosides such as GSLs, producing different metabolites (such as isothiocyanates (ITCs), thiocyanates, epiothionitriles, and nitriles) depending on the type of GSL and environmental conditions [64]. Normally in plants, GSLs are physically separated from endogenous myrosinase; however, when plant tissue is damaged (caused by insects or herbivory or during food preparation), the enzyme encounters these compounds, and enzymatic hydrolysis occurs immediately [65,66]. The breakdown of GSLs results in the generation of mainly ITCs, which are more bioactive. However, ITCs derived from indolic GSLs are unstable and spontaneously decompose to indole-3-carbinol (I3C) and 3,3′-diindolylmethane, among others [67,68].

The levels of GSLs vary in the different botanical parts of broccoli. This is due to their biosynthesis, which starts in the leaves and is transported from there to the rest of the organs, and which is more active in young growth stages and less so in mature stages [69]. Regarding the overall content of GSLs, sprouts present higher concentrations of these compounds (up to 162.19 µmol/g DW) than florets, stalks, and leaves (Table 3). In addition, broccoli sprouts also present higher levels of total GSLs than cauliflower sprouts (50.66 µmol/g DW) and even than cabbage sprouts (up to 91.94 µmol/g DW) [70]. Concerning the rest of the organs, florets also have important levels of GSLs (up to 25 µmol/g DW), followed by leaves and finally stalks with the lowest levels. The profile of these compounds also differs among the different botanical parts of broccoli (Table 3). The main GSLs present in broccoli florets were neoglucobrassicin, glucobrassicin, and glucoraphanin [71]. On the other hand, broccoli stalks and leaves presented similar profiles among them, highlighting glucoraphanin, neoglucobrassicin, and glucoiberin [10]. Finally, in the sprouts, the most abundant GSLs were glucoraphanin and glucoerucin [70].

According to Ilahy et al. [72], these BACs can be affected by both preharvest (e.g., genotype, environment, developmental stages, and farming practices) and postharvest (e.g., storage, processing, and packaging) effects. Genotype, organic farming, ultraviolet light, and ultrasound are some of the factors that positively affect these compounds. On the other hand, there are other factors such as high salinity, heat treatment, and domestic processing that can decrease their concentrations. The type of cooking is an influential factor in the concentrations of these compounds. In a review by Soares et al. [73], the effects of various cooking methods—steaming, boiling, frying, and microwaving—were examined, with the authors concluding that steaming was the most effective for preserving GSLs due to its lower loss of soluble matter.

ITCs are the most important breakdown products of GSLs and are even more bioactive than them. Within this group, sulforaphane (SFN) stands out, whose concentrations also differ among the botanical parts of broccoli (Table 3), being more abundant in the sprouts (up to 1483 µg/g DW) [33] and less in the leaves (up to 64 µg/g DW) [74]. The elevated levels in the sprouts result from their high content of glucoraphanin, the inactive precursor of SFN [75]. The concentrations of these compounds can be affected by certain factors, such as the cooking and drying methods. Processing temperature is one of the most crucial factors affecting the conversion of GSLs to ITCs by the enzyme myrosinase [37].

#### 3.3.2. Carotenoids

Carotenoids are lipophilic pigments found in plants, fungi, algae, and bacteria that must be obtained from food or supplements as humans cannot synthesize them [76]. These compounds have been linked to antioxidant and provitamin A activity, reducing the risk of diseases such as cancer, CVD, age-related macular degeneration, and photosensitivity associated with UV exposure [77,78].

Carotenoids are widely present in all botanical parts of broccoli, standing out especially in leaves with 1095.0 µg/g DW, being significantly higher than in stalks (15.6 µg/g DW) (Table 3). Lutein and neoxanthin were the predominant carotenoids in florets, stems, leaves, and sprouts. On the other hand, β-carotene was also one of the most predominant in florets and leaves, while it was not observed in stalks [10,79]. A previous study by dos Reis et al. [80] found that the cooking method affects these compounds, with higher concentrations of carotenoids, particularly lutein, being observed when various cooking methods (boiling, steaming, microwaving, and sous-vide) were applied to broccoli compared with fresh broccoli.

#### 3.3.3. Phenolic Compounds

PCs are another important bioactive compound present in broccoli. These compounds are secondary metabolites present in plants with a common structure: one or more phenolic groups attached to an aromatic or aliphatic structure. These compounds stand out for their role as antioxidants, although they have also been associated with other biological activities such as anti-inflammatory, anti-proliferative, antimicrobial, anticarcinogenic, anti-aging, and anti-thrombotic activities, among others [17,81].

Total polyphenol content (TPC) was up to 77.19 mg gallic acid equivalent (GAE)/g DW in the sprouts, being higher than in leaves, florets, and stalks, with 24.35, 10.74, and 9.39 mg GAE/g DW [33,82]. In the case of sprouts, they were found to have higher levels of polyphenols than sprouts of other species such as radish, lentils, beets, and amaranth [79]. On the other hand, polyphenols are compounds that are affected by external factors, especially thermal processes. Zhang et al. [83] demonstrated that when broccoli florets are subjected to elevated temperatures for a short period of time (especially stir-frying), the TPC can increase due to the inactivation of polyphenol oxidase.

A great diversity of phenolic acids (PAs) was identified and quantified in the different botanical parts of broccoli, especially in florets and sprouts (Table 3). Regarding their profile, iso-chlorogenic acid (59.85 mg/100 g DW) was the predominant acid in florets, while in sprouts it was led by sinapic acid (140.53 mg/100 g DW) [84]. On the other hand, a combination of caffeoyl, feruloyl, coumaroyl, and sinapoyl derivatives has been determined in the stalks [84]. Costa-Pérez et al. [85] also determined that the phenolic acid profile in the stalks varies depending on whether the stalks are intact or separated into heart and bark, with the latter having by far the lowest concentrations. Nevertheless, the PAs in leaves have been less quantified.

Flavonoids are another type of polyphenol present in broccoli. Higher levels of these compounds (up to 9.93 mg EC/g DW) have been identified in leaves than in florets (up to 6.33 mg EC/g DW) [59]. Kaempferol and quercetin were the main flavonoids identified in broccoli, with higher concentrations in leaves (up to 274.30 and 87.70 µg/g FW, respectively) than in florets [54]. In addition, these compounds have been shown to be antioxidant and anti-inflammatory agents in the human diet associated with a reduced risk of severe chronic diseases. However, they are also affected by cooking, decreasing at elevated temperatures [86].

**Table 3:** BACs of diverse botanical parts of broccoli.

Composition	Unit	Florets	Stalks	Leaves	Sprouts	Ref.
Organosulfur compounds						
Total GSLs	(µmol/g DW)	5.86–24.95	7.45	10.08	0.95–162.19	[10,70,71]
	(µmol/g FW)	-	-	-	4.02–45.60	[70]
Aliphatic GSLs						
Glucoalyssin	(µmol/g DW)	-	-	-	0.07	[70]
Glucobrassicanapin	(µmol/g DW)	-	-	-	0–0.11	[70]
	(µmol/g FW)	-	-	-	0–1.44	[70]
Glucoerucin	(µmol/g DW)	0.01–6.27	0.89	0.04	0.02–123.67	[10,70,71]
	(µmol/g FW)	-	-	-	0–6.09	[70]
Glucoiberin	(µmol/g DW)	0–0.88	0.97	0.65	0–13.90	[10,70,71]
	(µmol/g FW)	-	-	-	0–0.60	[70]
Glucoiberverin	(µmol/g DW)	-	-	-	0.14–6.26	[70]
Glucoibervirin	(µmol/g DW)	-	-	-	1.59	[70]
Gluconapin	(µmol/g DW)	0–2.73	0.03	0.04	0.04–2.00	[10,70,71]
	(µmol/g FW)	-	-	-	0.02–3.44	[70]
Glucoraphanin	(µmol/g DW)	0.14–14.97	3.79	2.77	0.05–43.60	[10,70,71]
	(µmol/g FW)	-	-	-	0–33.88	[70]
Progoitrin	(µmol/g DW)	0–4.54	0.24	0.02	0–28.45	[10,70,71]
	(µmol/g FW)	-	-	-	0.11–0.19	[70]
Sinigrin	(µmol/g DW)	0–3.16	<0.1	<0.1	0–15.00	[10,70,71]
	(µmol/g FW)	-	-	-	0–0.04	[70]
Aromatic GSLs						
Gluconasturtiin	(µmol/g DW)	0–0.44	0.02	0.11	0.13–14.90	[10,70,71]
Glucotropaeolin	(µmol/g DW)	0–0.04	-	-	-	[71]
Indolic GSLs						
4-Hydroxyglucobrassicin	(µmol/g DW)	0.01–3.29	0.07	0.26	1.10–5.30	[10,70,71]
	(µmol/g FW)	-	-	-	0–1.92	[70]
4-Methoxyglucobrassicin	(µmol/g DW)	0.01–3.92	0.16	0.17	0.80	[10,70,71]
	(µmol/g FW)	-	-	-	0.55	[87]
Glucobrassicin	(µmol/g DW)	0.10–27.69	0.10	0.24	0.04–3.20	[10,70,71]
	(µmol/g FW)	-	-	-	0–0.58	[70]
Neoglucobrassicin	(µmol/g DW)	0.02–45.95	1.11	5.78	1.50–10.71	[10,70,71]
	(µmol/g FW)	-	-	-	0.05–0.79	[70]
ITCs						
Erucin	(µmol/100 g DW)	2.85	-	-	-	[88]
Sulforaphane	(µg/g DW)	310.00–454.00	461.00–506.00	18.00–64.00	336.32–1483.76	[33,74,89]
	(µmol/100 g DW)	56.74	-	-	-	[88]
	(µmol/100 g FW)	-	-	-	57.00–58.00	[90]
Other GSL degradation products						
3,3-Diindolymethane	(mmol/100 g FW)	3.78	-	-	-	[91]
Indole-3-cabinol	(µmol/100 g FW)	-	-	-	1.00–2.00	[90]
	(mmol/100 g FW)	27.83	-	-	-	[91]
Carotenoids						
Total carotenoids	(μg/g DW)	181.00	15.60	1095.00	451.70	[10,79]
(α + β)-Carotene	(μg/g DW)	-	-	-	23.30	[79]
β-Carotene	(μg/g DW)	30.60	0.00	248.40	-	[10]
Lutein	(μg/g DW)	85.50	10.80	484.10	193.20	[10,79]
Neochrome	(μg/g DW)	-	-	-	10.60	[79]
Neoxanthin	(μg/g DW)	30.20	4.80	156.20	56.40	[10,79]
Violaxanthin	(μg/g DW)	34.70	0.00	206.30	37.20	[10,79]
Zeoxanthin	(μg/g DW)	-	-	-	23.50	[79]
Other carotenoids	(μg/g DW)	-	-	-	107.60	[79]
Phenolic compounds						
PAs						
Caffeic acid	(mg/100 g DW)	1.55	-	-	nd	[84]
Chlorogenic acid	(mg/100 g DW)	nd	-	-	37.26	[84]
Ferulic acid	(mg/100 g DW)	nd	-	-	73.85	[84]
Gentisid acid	(mg/100 g DW)	nd	-	-	80.80	[84]
Iso-chlorogenic acid	(mg/100 g DW)	59.85	-	-	nd	[84]
p-Coumaric acid	(mg/100 g DW)	nd	-	-	27.75	[84]
Sinapic acid	(mg/100 g DW)	3.43	-	-	140.53	[84]
Caffeoyl derivatives						
5-caffeoylquinic acid	(mg/g DW)	-	4.30	-	-	[85]
Caffeoyl derivative	(mg/g DW)	-	1.73	-	-	[85]
Caffeoyl-hexose derivative	(mg/g DW)	-	1.87	-	-	[85]
Di-caffeoylquinic acid derivative	(mg/g DW)	-	1.76	-	-	[85]
Feruloyl derivatives						
3-O-feruloylquinic acid	(mg/g DW)	-	0.82	-	-	[85]
Feruloyl-caffeoyl derivative	(mg/g DW)	-	6.45	-	-	[85]
Coumaroyl derivatives						
p-coumaroylquinic acid	(mg/g DW)	-	0.55	-	-	[85]
Sinapoyl derivatives						
1-Di-sinapoyl-2-feruloyl-gentiobioside	(mg/g DW)	-	0.70	-	-	[85]
1-Di-sinapoyl-2-feruloyl-gentiobioside (isomer)	(mg/g DW)	-	1.25	-	-	[85]
1,2′-Di-sinapoyl-2-feruloyl-gentiobioside	(mg/g DW)	-	1.94	-	-	[85]
1,2,2′-Tri-sinapoyl-gentiobioside	(mg/g DW)	-	12.70	-	-	[85]
Di-sinapoyl-diglucose	(mg/g DW)	-	2.26	-	-	[85]
Di-sinapoyl-gentiobioside I	(mg/g DW)	-	0.70	-	-	[85]
Di-sinapoyl-gentiobioside II	(mg/g DW)	-	4.69	-	-	[85]
Sinapoyl-gentibioside	(mg/g DW)	-	0.85	-	-	[85]
Sinapoyl hexoside	(mg/g DW)	-	0.59	-	-	[85]
Flavonoids						
Total flavonoids	(mg QE/g DW)	-	2.20–8.10	-	75.52–117.26	[33,89]
	(mg CE/g DW)	2.84–6.33	-	7.84–9.93	3.18	[59,92]
Flavonols						
Total flavonols	(mg CE/g DW)	-	-	-	0.19	[92]
Kaempferol	(µg/g FW)	0.80–87.70	-	108.00–274.30	-	[54]
Quercetin	(µg/g FW)	1.80–29.00	-	8.00–32.20	-	[54]
Polyphenols						
Total Polyphenols	(mg GAE/g DW)	10.74	9.39	24.35	35.90–77.19	[33,82]

FW: fresh weight; DW: dry weight; nd: not detected; ITCs: isothiocyanates; PAs: phenolic acids; QE: quercetin equivalent; CE: catechin equivalent; GAE: gallic acid equivalent.

## 4. Biological Properties and Beneficial Effects on Health

The BACs present in all botanical parts of broccoli confer properties associated with the prevention and treatment of diseases, such as anti-tumor, anti-inflammatory, antimicrobial, antioxidant, regulators of metabolic syndrome, and cardioprotective and neuroprotective agents (Table 4).

### 4.1. Anti-Cancer Activity

With an estimated 10 million deaths in 2020, cancer is one of the leading causes of death worldwide. In recent years, the most common cancers have been breast, lung, colorectal, and prostate cancer [93]. Recently, there has been an increased interest in investigating the anti-cancer potential of raw vegetables, such as the *Brassicaceae* family, rich in ITCs, polyphenols, and vitamins, with the intention of finding alternatives to current therapies that produce numerous side effects [61,94,95].

The antitumor effect of the broccoli has been associated with the cleavage products of GSLs, mainly SFN and I3C [96,97,98,99]. These compounds can inhibit tumorigenesis and proliferation of cancer cells and induce apoptosis in several types of cancer, such as breast, prostate, lung, and colorectal cancer, modulating multiple cellular pathways responsible for cancer development and progression [100]. Its related molecular mechanisms include upregulation of the p53 tumor suppressor gene that upregulates Bax and cleaved caspase-3, inhibition of the activator of transcription (STAT), and regulation of the cyclin-dependent kinase CDK2 [101,102]. In addition, Zang et al. [103] have reported that SFN interferes with the RAF/MEK/ERK signaling pathway preventing the formation of active stress fibers and preventing breast cancer cell metastasis. Numerous investigations in cell models, murine models, and clinical studies have demonstrated the anticarcinogenic activities of broccoli and its by-products [37,104,105].

At in vitro level, Le et al. [59] compared the cytotoxic activity of methanolic extracts of florets, leaves, and seeds. They observed that seed extracts exerted greater cytotoxicity against lung (A549), colorectal (Caco-2), and hepatocellular (HepG2) cancer cell lines than the other extracts due to their higher glucosinolate content [106]. Although the antitumor activity of broccoli is high, it can be diminished by the degradation of the BACs responsible for it. For this reason, Radünz et al. [107] carried out electrospray encapsulation of broccoli extracts and compared their cytotoxic activity against mouse glioma cells (GL261), being more selective and effective in the encapsulated extracts than in the non-encapsulated ones. In another in vitro study by Cao et al. [108], administering broccoli extracellular vesicles (BEVs) with the drug 5-fluorouracil (5-FU) on colorectal cancer cells (HT-29) induced apoptosis by stimulating reactive oxygen species (ROS) production and altering mitochondrial function. In addition, broccoli vesicles were able to reverse the resistance generated by cancer cells to the drug.

On the other hand, the possible effect of maternal/prenatal administration of dietary broccoli sprouts has also been investigated in mouse models of breast cancer (HEr2/neu), where it was observed that they can profoundly suppress ER-negative mammary tumorigenesis in the offspring through modulation of histone acetylation and DNA methylation status, as well as tumor-related gene expression [109]. In another study by Ho et al. [110] with murine models of colorectal cancer (male Balb/c mice), the effects of a broccoli diet with *Escherichia coli* modified to bind to the surface of cancer cells and secrete myrosinase converting GSLs to SFN were studied. This increase in SFN, a compound more bioactive than GSLs, resulted in a significant 75% reduction in colorectal cancer. Human studies have also demonstrated the anti-cancer activity of broccoli and its by-products. In patients with melanoma, the ingestion of capsules containing broccoli sprout extracts produced a decrease in plasma pro-inflammatory cytokines, as well as an increase in decorin, a tumor suppressor [111]. Likewise, in another study, broccoli soup rich in glucoraphanin was given to men with prostate cancer, showing an attenuating effect on the expression of oncological pathways [112].

### 4.2. Anti-Inflammatory Activity

The process of inflammation, although a defense mechanism of the immune system, can become detrimental to health and contribute to various diseases if it becomes chronic [113]. Herbs and vegetables, due to their high content of phytochemicals, antioxidants, and other BACs, have been investigated as positive inflammation-reducing agents [100]. Numerous research studies have shown that broccoli and its by-products contain phytochemicals such as SFN, I3C, ITCs, and flavonoids that contribute to its anti-inflammatory properties. The main mechanisms of action associated with these compounds are a reduction in the production of pro-inflammatory substances such as cytokines (interleukin-6 and tumor necrosis factor-alpha (TNF-α)) and prostaglandins [114,115]. SFN is also considered an immunomodulatory molecule because of its ability to influence the activity of immune cells involved in inflammation, such as macrophages and lymphocytes [116,117,118]. On the other hand, the flavonoid quercetin also contributes to the control of the inflammatory response by modulating pathways involved in the inflammatory process (nuclear factor kappa B (NF-κB)) [119].

At the in vitro level, Ferruzza et al. [120] studied the effects of 5-day-old broccoli sprout juice on TNF-alpha-stimulated Caco-2 cells, also using elicitation to improve the nutraceutical content of the sprouts. As a result, they found that elicitation led to an increase in PCs in the sprouts (especially several anthocyanins), and this translated into an increased protective effect on the intestinal barrier integrity of the inflamed cells compared to the control juice. Another study using lipopolysaccharide (LPS)-stimulated HepG2 cells demonstrated the anti-inflammatory potential of SFN in broccoli, as it suppressed IL-6 transcription and secretion and consequently reduced secretion of the inflammatory hormone hepcidin [121].

The anti-inflammatory activity of broccoli and its derived products has also been evidenced in animal and clinical studies. In a mouse model, the effects of SFN on inflammation in hemorrhagic shock/resuscitation using male C57/BL6 mice were studied, and it was found that this compound not only exerts protective effects but also systematic effects via down-regulation of pro-inflammatory cytokines [122]. Furthermore, another murine model suggested that the anti-inflammatory effect of broccoli florets after microwave heating (3 min) was comparable to that of raw florets in terms of parameters such as colon length and lesion severity, as well as in the decrease in IL-6 [123]. Finally, in an intervention study, feeding 30 g of fresh broccoli sprouts daily to overweight people led to a significant reduction in IL-6 and C-reactive protein (CRP) levels, highlighting their anti-inflammatory activity [124].

### 4.3. Antioxidant Activity

As mentioned above, broccoli and its different botanical parts contain several BACs such as glucoraphanin, SFN, PCs, and carotenoids that provide antioxidant activity. Antioxidants help protect cells from damage caused by free radicals, unstable molecules that can cause oxidative stress and contribute to various diseases [125]. Broccoli SFN, in addition to directly counteracting free radicals, can also induce phase II antioxidant enzymes [100].

The various in vitro assays of antioxidant activity have shown that it varies depending on the various parts of broccoli. In addition, there are other factors that may influence antioxidant activity, such as gastrointestinal digestion. In an in vitro simulated digestion process, de la Fuente et al. [60] found that this process reduced the antioxidant capacity of broccoli sprouts by 52% in the ORAC assay. In addition, Lv et al. [126] found that germination also affects antioxidant capacity, as the antioxidant activity of broccoli sprouts increased significantly after 3 days of germination due to the accumulation of phytochemicals, such as PCs, during germination. On the other hand, cell model-based studies have also shown that SFN can attenuate oxidative stress by purging excess ROS and activating detoxification pathways via lysosomal Ca^2+^ release and subsequent nuclear translocation of EB transcription factor [127].

Animal models have also been used to evaluate the antioxidant properties of broccoli; for example, Cardenia et al. [128] used commercial broccoli extract capsules in female mice subjected to exhaustive exercise, and Xu et al. [129] used floret powder as well as glucoraphanin from seeds in C57BL/6 mice on a high-fat diet (HFD). Both studies showed the activities of endogenous antioxidant enzymes such as superoxide dismutase, glutathione-S-transferase, catalase, and glutathione reductase increased with broccoli and its compounds, and thus oxidative stress also decreased.

### 4.4. Antimicrobial Activity

Many studies demonstrate that broccoli and its derived compounds, such as GSLs and ITCs, exert antimicrobial activities. ITCs have been shown to reduce microbial growth by altering several cellular mechanisms, such as inhibiting enzyme function, altering enzyme structure, and increasing oxidative stress [130]. SFN has been shown to have antibacterial activity against *Helicobacter pylori*, a bacterium associated with peptic ulcers and gastrointestinal infections [131]. I3C and PCs have also been associated with inhibitory activity against strains of *Escherichia coli* and *Staphylococcus aureus* [100]. Some compounds, such as 3,3′-diindolimethane, may even act as inhibitors of biofilm formation and reduction in bacterial load [132].

Accordingly, an in vitro study was carried out with extracts of broccoli florets, leaves, and seeds, all of which showed antimicrobial activities against foodborne pathogens such as *Staphylococcus aureus*, *Bacillus subtilis*, *Salmonella typhimurium*, and *Escherichia coli*. The most potent extracts with inhibitory activity were those from leaves and florets, and leaves achieved the lowest minimum inhibitory concentrations [59]. In another study with pathogenic bacteria (*Bacillus cereus, Staphylococcus xylosus*, and *Shigella flexneri*, among others), phytopathogenic fungi (*Aspergillus niger* and *Collectotrichum gloeosporioides*), and yeasts (*Candida albicans* and *Rhodotorula* sp.), extracts of broccoli florets and stalks showed inhibitory effects against all of them, especially against *Staphylococcus xylosus*. In addition, the antimicrobial components in crude extracts were thermoresistant, and the highest activity was observed under acidic conditions [133]. Broccoli sprout extracts have also shown antimicrobial activity against different bacterial strains in vitro, showing a strong correlation with their organic acid content [134]. Furthermore, Abukhabta et al. [135] found that the antimicrobial activity of broccoli can be increased by cooking and to a greater extent when mustard seeds are added, becoming comparable to that of some antibiotics such as gentamicin against strains of *E. coli* and *S. typhimurium*. Finally, the antimicrobial effect of broccoli sprouts in humans was evaluated in a previous study in which patients infected with *Helicobacter pylori* were given broccoli sprout powder rich in SFN (>22.5 μmol/g). After consuming this powder for 28 consecutive days, the patients experienced a decrease in their serum nitric oxide levels [136].

### 4.5. Other Biological Activities

Broccoli and its derived products have also been proven in numerous studies to possess other biological activities such as regulation of metabolic syndrome, among others.

Metabolic syndrome is a group of metabolic disorders that includes conditions such as insulin resistance, hypertension, central obesity, and atherogenic dyslipidemia, which can lead to diabetes and CVD [137]. Several types of studies have indicated that broccoli and its derivatives can regulate these types of pathologies by suppressing adipogenesis and gluconeogenesis, with nuclear factor erythroid 2-related factor 2 (Nrf2) and AMPKc being the main targets. A study by Lee et al. [138] investigated the effects of sinigrin on adipogenesis in 3T3-L1 mouse preadipocytes and found that it inhibited this process by inducing cell cycle arrest. Similarly, studies in animal models have shown that administration of broccoli extracts can decrease the expression of genes responsible for lipogenesis, fasting blood glucose, insulin resistance, body weight, and atherogenic index and increase the expression of genes contributing to fatty acid oxidation [139,140]. Finally, in obese patients with type 2 diabetes mellitus (T2DM), SFN from broccoli sprouts has been shown to lower fasting blood glucose and reduce glycosylated hemoglobin (Hba1c), with a greater effect in patients with high plasma triglyceride concentrations [141].

Neurodegenerative diseases include Alzheimer’s disease, Parkinson’s disease, Huntington’s disease, anxiety, and spinocerebellar ataxia, among others, which have neuropathological symptoms associated with oxidative stress and inflammation [142]. On the other hand, broccoli has also been associated with neuroprotective effects against this type of pathology, due to its ability to activate the redox mediator Nrf2. Some in vitro studies using C6 astrocyte cell lines and monocytes from children with autism spectrum disorders (ASD) have suggested that SFN from broccoli may reverse the increase in oxidative stress by increasing enzymatic antioxidants and reducing the production of pro-inflammatory factors (TNF-α, IL-1β) through modulation of Nrf2 [143,144]. Similarly, broccoli and its by-products have been shown to have neuroprotective effects in mice, with broccoli leaves improving cognitive function in mice, as well as having antioxidant effects and inhibiting brain acetylcholinesterase [145]. In addition, recent research has highlighted the neuroprotective effects of I3C, as its chronic administration for 21 days in LPS-treated male Wistar rats produced an improvement in LPS-induced motor function, oxidative damage, and neuroinflammation through inhibition of NF-κB signaling [146].

In addition to the health benefits mentioned above, broccoli and its compounds have also been associated with benefits for heart health, bone health, eye health, gastrointestinal health, and the immune system due to its high content of minerals, vitamins, flavonoids, ITCs, carotenoids, indoles, and especially SFN [1,100,147].

**Table 4:** Biological properties and beneficial effects on health.

Botanical Part	Type Experiment	Experiment Details	Results	Ref.
Anti-cancer activity				
Florets, leaves, and seeds (ethanol, methanol, and hot water extracts)	In vitro: HepG2, Caco-2, and A549 cells	Cytotoxic assay, cell cycle analysis, and MMP	Seed extracts with the strongest cytotoxicity	[59]
			↑ Apoptosis, subG1 phase	
			↓ G0//G1 phase, G2/M phase	
			↓ MMP level significantly	
Florets (encapsulated ethanol/water extracts)	In vitro: GL261 mouse cell line	Cell viability analysis	Selective activity against tumor cells since they did not alter the viability of astrocytes	[107]
			Antiglioma effect at all concentrations tested	
Edible parts (BEVs)	In vitro: HT-29 cells	Viability assay, cell apoptosis analysis, analysis of the synergistic effect of the combination of BEVs and 5-FU, cell cycle analysis, plate colony formation assay, cell scratch assay, ROS detection, MMP detection,	BEVs cytotoxic to colorectal cells	[108]
			↑ Inhibition of HT-29 cell viability	
			S-phase arrest and proliferation inhibition	
			↑ Pro-apoptotic Bax and Caspase-3 mRNA	
			↓ Anti-apoptotic Bcl-2 levels	
			↑ Intracellular ROS levels	
			Reversion of 5-FU resistance by modulating the PI3K/Akt/mTOR pathway	
Sprouts (diets containing them)	In vivo: Her2/neu mammary tumor female mouse model	Cell viability assay, histone acetyltransferase activity assay, global DNA methylation, and hydroxymethylation analysis	↓ Mammary cancer formation in the nontreated mouse offspring	[109]
			Suppressive effects on mammary cancer in adult mice, not as profound as the maternal broccoli sprout diet preventive effects	
			↑ Transcription levels of p16 and p53, tumor suppressor genes	
			↓ Bmi 1 (tumor-promoting gene), methyltransferases, histone deacetylases	
Not specified (broccoli diet)	In vivo: Balb/c mice, colorectal cancer model	Broccoli diet with engineered commensal *E. coli*	↑ Tumor regression	[110]
			75% tumor reduction in the colorectal region	
			↑ Conversion of GSLs to SFN	
Sprouts (gel capsules with extracts)	In vivo: Clinical trial with melanoma patients	Three dosage groups received 50, 100, and 200 μmol of oral broccoli sprout extract once daily for 28 days.	Dose–response relationship in SFN levels	[111]
			↓ Plasma levels of pro-inflammatory cytokines	
			↑ Decorin, tumor suppressor	
Not specifies (broccoli soup)	In vivo: Clinical trial with men with low- or intermediate-risk prostate cancer.	Consumption of a 300 mL portion of broccoli soup per week for 12 months	↓ Gene expression changes and associated oncogenic pathways	[112]
			Inverse association between consumption of cruciferous vegetables and cancer progression	
Anti-inflammatory activity				
Sprouts (juice)	In vitro: TNF-α-stimulated Caco-2 cells	Measure of monolayer integrity and experimental intestinal cell model	↓ Trans-epithelial electrical resistance	[120]
			Intestinal cell protection is positively correlated with procyanidin B2, cryptochlorogenic acid, neochlorogenic acid, quercetin-3-glucoside, cinnamic acid, and five different cyanidine-3-glucosides	
Not specified (SFN from broccoli)	In vitro: HepG2 cell line	Measuring IL-6 cytokine protein secretion and gene expression, hepcidin protein secretion, and cell viability assay	↓ IL-6 gene expression, hepcidin secretion	[121]
			No toxic effect in the treatment of HepG2 cells with SFN	
Not specified (SFN from broccoli)	In vivo: Male C57/BL6 wild-type mice and transgenic ARE-luc mice	Fluid resuscitation performed via intraperitoneal administration of SFN	Hemorrhagic shock/resuscitation associated with pulmonary Nrf2 activation	[122]
			↑ Pulmonary Nrf2 activity, alveolar macrophage activation	
			↓ Lung damage, systemic pro-inflammatory mediators	
Florets (raw and lightly cooked broccoli)	In vivo: Male C57BL/6 mice with dextran sulfate sodium-induced colitis	Three groups with control, raw broccoli, or lightly cooked broccoli diet for 14 days	Alleviated the clinical symptoms of colitis	[123]
			↓ Weight loss, stool formation, fecal bleeding, combined disease activity index, colon lesions	
			↑ Colon length	
			↓ IL-6, CCR2, VCAM-1	
Sprouts (included in the diet)	In vivo: Clinical trial with overweight subjects	Daily consumption of 30 g of raw or fresh broccoli sprouts for 10 weeks	↓ IL-6 and CRP significantly	[124]
			No significant changes in weight or body mass index	
Antioxidant activity				
Sprouts (rehydrated freeze-dried samples)	In vitro	In vitro gastrointestinal digestion, BACs, and antioxidant capacity assays performed	↓ ORAC and TEAC levels after simulated digestion	[60]
			Antioxidant capacity retained bioaccessible fraction	
Seeds and sprouts (methanol extracts)	In vitro	Measurement of total phenolic and flavonoid contents in sprouts on different germination stages	Maximum SFN, TP, and TF contents in sprouts on day 3	[126]
			Higher antioxidant activity in broccoli sprouts than in seeds	
			After in vitro digestion, higher values of DPPH and FRAP in sprouts than in seeds	
Not specified (SFN from broccoli)	In vitro: HeLa, HepG2, 1321N1, HEK293, and human fibroblast cells	Autophagic flux and lysosome biogenesis studies, ROS and Ca^2+^ imaging	Induction of a TFEB nuclear translocation via a Ca^2+^-dependent but mTOR-independent mechanism through a moderate increase in ROS	[127]
			↑ Expression of autophagosome and lysosome biogenesis genes	
Unknown (commercial broccoli extract capsules)	In vivo: Female Wistar rats subjected to exhaustive exercise	Four diverse groups were fed a standard diet with or without broccoli extract for 45 days	Exhaustive exercise was responsible for tissue damage	[128]
			↓ LDH and oxysterols	
			↑ GST, GR, CAT	
Florets and seeds	In vivo: HFD-fed C57BL/6 mice	Mice with HFD containing 18.77 g/kg body weight freeze-dried broccoli powder or 150 μmol/kg body weight glucoraphanin	↓ Liver weights and adipose tissue masses, concentrations of serum inflammatory factors	[129]
			Alleviated HFD-induced oxidative stress, ↓ MDA	
			↓ SOD and CAT activity	
Antimicrobial activity				
Florets, leaves, and seeds (ethanol, methanol, and hot water extracts)	In vitro: Gram-negative and Gram-positive pathogenic bacteria	Bacterial inhibitory activity was analyzed using the agar well diffusion and the broth microdilution techniques	Leaves and floret extracts presented stronger inhibitory activities against tested bacteria than seed extracts	[59]
			High inhibitory effects on *Bacillus subtilis*, *Salmonella typhimurium,* and moderate effects on *Staphylococcus aureus* and *Escherichia coli*	
			*Bacillus subtilis* was the most susceptive	
			Leaf extracts exhibited inhibitory activity with the lowest minimum inhibitory concentrations	
Florets and stalks (water extracts)	In vitro: Gram-negative and Gram-positive pathogenic bacteria, and phytopathogenic fungi and yeasts	Bacterial inhibitory activity was determined using the well diffusion assay	Inhibitory effects against Gram-positive and Gram-negative pathogenic bacteria (*Bacillus cereus*, *Staphylococcus xylosus*, *Staphylococcus aureus*, *Shigella flexneri*, *Shigella sonnei*, *Proteus vulgaris*), phytopathogenic fungi (*Colletotrichum gloeosporioides*, *Asperigillus niger*), and yeasts (*Candida albicans*, *Rhodotorula* sp.)	[133]
			*Staph. xylosus* was the most susceptible	
			Antibacterial activity proteinaceous in nature	
Sprouts (aqueous extracts)	In vitro: Five bacterial strains	Antimicrobial activity by broth microdilution method	Notable antimicrobial activity against *Escherichia coli* O 157: H7 ATCC 35150, *Salmonella typhimurium* ATCC 14028, *Listeria monocytogenes* ATCC 35152, *Bacillus cereus* ATCC 11778, and *Staphylococus aureus* ATCC	[134]
			Antimicrobial activity of broccoli extracts is like that of red cabbage and higher than that of Galega kale and Penca cabbage	
Florets (SFN extracts)	In vitro: Strains of the genera *Salmonella*, *Escherichia*, *Staphylococcus*, *Listeria*, and *Bacillus*)	Antimicrobial activity was tested using the disk diffusion method	Raw broccoli has antimicrobial activity only against *B. cereus*	[135]
			Cooked broccoli extracts showed considerable antimicrobial activity against the tested strains, being higher in those with added mustard seeds	
			Organosulfur compounds related to the antimicrobial activity	
			*B cereus* was the most susceptible	
			Lower antimicrobial activities for the Gram-positive bacteria compared to the Gram-negative ones	
			Effect comparable to that of some antibiotics	
Sprouts (powder)	In vivo: Clinical trial with *Helicobacter pylori*-infected patients	Consumption of broccoli sprout powder (22.5 µmol SFN/g) for 28 days	↓ serum nitric oxide (NO) metabolites	[136]
Other biological activities				
Not specified (sinigrin from broccoli)	In vitro: 3T3-L1 mouse preadipocytes	Analysis of the effects of sinigrin on adipogenesis and its underlying mechanisms	↓ Expression of C/EBPα, PPARγ, leptin, and aP2	[138]
			Cell arrest in the G_0_/G_1_ phase	
			↑ Expression of p21 and p27, phosphorylation of AMPK, MAPK, and ACC	
			Suppression of production of CDK2, pro-inflammatory cytokines	
Not specified (juice)	In vivo: Male C57BL/6 J mice, type 2 T2DM	Administration of broccoli juice via gavage for 18 weeks	↓ Fasting blood glucose, insulin resistance, levels of TC, TG, LDL-c, and MDA	[139]
			Regulate lipid metabolism	
			↓ Relative abundance of genus *Allobaculum* and families *Odoribacteraceae*, *Rikenellaceae*, and *S24-7*	
			↑ Relative abundance of the genera *Odoribacter* and *Oscillospira* and the families *Erysipelotrichaceae* and *Rikenellaceae*	
Not specified (ethanolic broccoli extract)	In vivo: *Caenorhabditis elegans*, HFD-induced male Wistar rats	Supplementation of broccoli extract for 10 weeks	↓ Fat content, body weight gain, food efficiency, atherogenic index of plasma	[140]
			↓ Adipogenesis-related transcription factors (*Cebpa*, *Srebf1*, *Pparg*) and lipogenic genes (*Fasn*, *Adipoq*)	
			↑ Oxidative enzyme-encoding genes (*Acox1*, *Acot8*)	
			↓ Fatty acid transport and synthesis-related genes (*Fasn*, *Fatp4*, and *Srebf1*)	
			Improvement of glucose tolerance	
Sprouts (extracts with 150 µmol SFN per dose)	In vivo: Clinical trial with obese patients with dysregulated T2DM	Ingestion of SFN-rich broccoli sprout extract for 12 weeks	↓ Fasting glucose, HbA1c, gluconeogenesis-related enzymes	[141]
Unknown (SFN extracts)	In vitro: C6 astrocyte cell line	Analysis of the potential mechanisms involved in the glioprotective effects of SFN	Activation of NF-κB and hypoxia-inducible factor-1α	[143]
			Modulation of the expression of the Toll-like and adenosine receptors	
			↑ Nrf2 and HO1	
			Modulation of superoxide dismutase activity and glutathione metabolism	
Unknown (SFN extracts)	In vitro: Monocytes of children with ASD	Evaluation of Nrf2 expression/activity along with parameters of inflammation and nitrative stress	↓ NF-κB signaling, LPS-induced effects on nitrative stress	[144]
			↓ Oxidative stress and inflammation	
			SFN protects against nitrative stress and inflammation	
Leaves (chloroform fraction)	In vitro: PC12 cells In vivo: Aβ-induced mice	Examination of the antiamnesic effects of broccoli leaves in vitro and in vivo tests on Aβ-induced neurotoxicity	Inhibition against AChE	[145]
			↑ Cognitive function	
			↓ Oxidative stress and AChE activity	
			Natural resource for ameliorating Aβ_1–42_-induced learning and memory impairment	
Unknown (I3C extracts)	In vivo: Male Wistar rats	Chronic administration of I3C for 21 days	Improvement in motor functions, coordination, learning, and memory	[146]
			↓ Inflammatory cytokines (TNF-α and IL-6), MDA	
			Inhibition of NF-κB	
			↑ Reduced glutathione, superoxide dismutase, and catalase	
			Neuroprotective effect of I3C via amelioration of LPS-induced behavioral alterations, oxidative damage, and neuroinflammation	

MMP: assessment of mitochondrial membrane potential; BEVs: broccoli extracellular vesicles; 5-FU: 5-Fluorouracil; ROS: reactive oxygen species; mTOR: mechanistic target of rapamycin kinase; GSLs: glucosinolates; SFN: sulforaphane; Nrf2: nuclear factor erythroid 2-related factor 2; CCR2: C-C chemokine receptor type 2; VCAM-1: vascular cell adhesion molecule 1; CRP: C-reactive protein; BACs: bioactive compounds; TEAC: Trolox equivalent antioxidant capacity; ORAC: oxygen radical absorbance capacity; DPPH: 2,2-diphenyl-1-picrylhydrazyl; FRAP: ferric reducing antioxidant power; TFEB: transcription factor EB; LDH: lactate dehydrogenase; GST: glutathione S-transferase; GR: glutathione reductase; CAT: catalase; HFD: high-fat diet; MDA: malondialdehyde; SOD: superoxide dismutase; NO: nitric oxide; C/EBPα: CCAAT/enhancer-binding protein α; PPARγ: peroxisome proliferator-activated receptor gamma; AMPK: MP-activated protein kinase; MAPK: mitogen-activated protein kinase; ACC: acetyl-CoA carboxylase; TC: total cholesterol; TG: triglycerides; LDL-c: low-density lipoprotein cholesterol; T2DM: type 2 diabetes mellitus; HbA1c: hemoglobin A1c; NF-κB: nuclear factor kappa-light-chain-enhancer of activated B cell; HO1: heme oxygenase-1; LPS: lipopolysaccharide; AchE: acetylcholinesterase; I3C: indole-3-carbinol, ↑: increase; ↓: decrease.

## 5. Novel Extraction Techniques of Bioactive Compounds

In recent years, the extraction processes of BACs have focused on the search for environmentally friendly solvents and the development of more ecological and sustainable techniques [148]. In addition, there is substantial evidence that the use of green technologies in species of the *Brassicaceae* family can increase the content and improve the extraction of BACs, which can then be applied in food matrices, promoting a circular economy [149]. These techniques allow the extraction of various BACs, such as GSLs, Pas, and flavonoids, as well as vitamins, minerals, and even DF, from the different botanical parts of broccoli [150].

Common green extraction techniques used to extract BACs from agricultural and food by-products include ultrasound-assisted extraction (UAE), microwave-assisted extraction (MAE), supercritical fluid extraction (SFE), and pressurized liquid extraction (PLE), including accelerated solvent extraction (ASE) [151,152,153,154]. Other techniques include pulsed electric field (PEF), ionic liquid extraction (ILE), and enzyme-assisted extraction (EAE) [155]. Table 5 summarizes these green extractions used for broccoli.

UAE is a technique that uses ultrasonic energy and solvents to create acoustic cavitation, whose bubbles generate shock waves and the accelerated collision between particles causes fragmentation of the cellular structure. Numerous studies have evaluated the extraction of compounds using this technique in broccoli florets, stems, and leaves with water or ethanol, resulting in elevated levels of antioxidant capacity, flavanols, GSLs, and SFN, as well as high-quality antimicrobial efficacy against species such as *Pseudomonas* and *Candida krusei* [82,156,157,158]. In addition, this technique yielded more BACs than SFE but less than PLE [159]. On the other hand, Martínez-Zamora et al. [160] recently found that the solid/liquid ratio influences the extraction of SFN and GSLs, and PCs can be influenced by the extraction time and ratio used.

MAE is another environmentally friendly extraction technique that has been widely used in recent years and is based on the selective heating of polar molecules with microwave energy, thus reducing extraction time, solvent consumption, and solvent residues [161]. Garcia et al. [152] applied this technique to broccoli florets, stems, and leaves using methanol as solvent at a maximum temperature of 75 °C, obtaining an improvement in phenolic yield (from 45 to 133% in leaves and florets, respectively) in less time than the maceration technique.

Finally, SFE has also been widely used with broccoli and its various parts. This is a technique that uses supercritical solvents with different physicochemical properties that, thanks to their low viscosity and high diffusivity, are a better transport medium than liquids, thus offering faster and more efficient extraction rates [162]. Supercritical fluid technology using CO_2_ has been applied to broccoli florets, leaves, and stems, obtaining high-quality extracts in terms of microbial efficiency, as well as high yields of compounds such as β-carotene, PCs, chlorophylls, and phytosterols, and high antioxidant capacity [13,35,156]. This technique has advantages over those mentioned above, such as its higher yield in the recovery of BACs and the low temperature required to avoid degradation of these compounds, but its extraction times and costs are counterproductive [149].

## 6. Food Application

Due to growing public concern about chronic diseases caused by dietary patterns, functional foods with health benefits have gained popularity [163]. Moreover, the revalorization of by-products from the food industry is important to reduce the substantial amounts of food waste currently generated [164]. The different botanical parts of broccoli have been reported to be used in the formulation of new functional food products to promote their nutraceutical and functional properties and as a strategy to reduce food waste [165]. The valorization of broccoli by-product extracts has become a promising possibility due to the high DF content of the stalks, as well as the GSL contents and the antioxidant properties of the leaves.

The usefulness of broccoli by-products to produce functional foods enriched with BACs has been widely described in various food matrices such as beverages, snacks, bread, or cakes [166,167,168,169]. However, the effects of adding broccoli extracts have been most studied in bakery products [149]. Currently, most of the bakery products are formulated with refined wheat flour and other ingredients of low nutritional value, so for this reason it is interesting to look for new ingredients that improve the nutritional quality of these products [170,171]. Cakes, biscuits, bread, pasta, and crackers are the bakery products studied for the replacement of the wheat flour with the different broccoli extracts (Table 6). These extracts are incorporated in the form of flours or powders obtained by drying (hot air or freeze-drying) the different botanical parts of broccoli, which allows them to maintain an adequate nutritional composition and physicochemical properties [172].

Several studies have used broccoli leaves in the form of powder to replace potato and corn starch in gluten-free (GF) mini sponge cakes, resulting in content of GSLs and AAs and TPC improvements [173,174,175]. In addition, these extracts also resulted in GF products with high firmness, elasticity, and adhesiveness, which are among the main textural problems of commercial GF products [176]. Khalaf et al. [177] also investigated the physicochemical characteristics and antimicrobial properties of low-calorie cake with the replacement of wheat flour with gamma-irradiated florets and leaf powder. The results, in addition to showing an increase in protein, lipids, DF, and PCs, also revealed a decrease in microbial count with increasing broccoli powder content.

The incorporation of broccoli floret leaves and stalks in bread has been widely described [178,179,180]. Baqar et al. [180] evaluated the physicochemical, rheological, and sensory attributes of bread using several proportions of floret flour reporting and increments of DF, protein, and TPC. Moreover, the concentrations of 1 and 3% of floret broccoli powder obtained an acceptable sensory evaluation without significantly altering the sensory characteristics of the bread. Comparable results were shown by Krupa-Kozak et al. [178], who replace 5% of corn starch with leaf powder in GF bread, resulting also in an improvement in the protein and PCs content. In addition, this reformulated GF bread also exhibited in two in vitro model systems (bovine serum albumin and BSA-methylglyoxal) inhibitory activity against advanced glycation end-products, compounds related to aging, and some chronic diseases such as diabetes [181].

Broccoli stalk powder has also been used to improve the nutraceutical potential of other bakery products such as biscuits and crackers. In biscuits, some studies have evaluated the replacement of wheat flour with broccoli stalk powder at high concentrations of up to 15%, obtaining products with higher levels of GSLs, carotenoids, and PCs, and even with a more cohesive and workable dough [182,183]. In addition, Sayem et al. [182] also compared the addition of broccoli stalk powder effects with banana peel or cauliflower stalk powders, resulting in higher overall acceptability of broccoli biscuits at 5% substitution. On the other hand, Lafarga et al. [184] also used broccoli stalk powder to make baked crackers formulated using a 12.5 and 15% flour substitution level, resulting in a product with higher DF and antioxidant capacity. In addition, after performing in vitro gastric digestion, they found that the TPC, FRAP, and DPPH scavenging capacities of the reformulated crackers had increased, demonstrating that a sustained release of BACs occurs when the broccoli cell wall is ruptured upon consumption of the crackers.

On the other hand, broccoli by-product extracts have also been used to prepare healthier pasta [185,186]. Bokić et al. [187] developed functional spaghetti containing 5, 10, and 15 g/100 g of broccoli powder with the intention of improving the nutritional profile of conventional pasta while maintaining sensory properties. In the uncooked pasta, there was an increase in protein, lipid, mineral, GSLs and synaptic acid derivative content, TPC, and antioxidant capacity. However, cooking slightly decreased TPC and synaptic acid derivatives but also increased flexibility. Broccoli BACs such as GSLs have low stability and are therefore degraded by cooking. This highlights the need to apply encapsulation techniques to broccoli extracts to prevent the degradation of these compounds and increase their stability and bioavailability [107,188].

Finally, according to the results presented in Table 6, the main effect of the addition of broccoli extracts in the new products is the change in color, being darker and greener. In addition, other organoleptic properties such as taste and texture may be negatively affected. These alterations are a crucial factor to consider and try to optimize to avoid poor consumer acceptance.

**Table 6:** Application of diverse botanical parts of broccoli in bakery products.

Botanical Part	Food Product	Formulation	Outcomes	References
Leaves	GF mini sponge cakes	Replacement of potato and corn starch with freeze-dried broccoli powder (0, 2.5, 5, 7.5%)	Increased GSLs and derivatives, TPC, and antioxidant capacity	[173,174,175]
			Dark green color, intense broccoli aroma, and flavor	
			Higher concentrations of free amino acids (FAAs), including EAAs	
			Increased instrumental firmness, maintenance of elasticity, mastication, and adhesiveness	
Florets and leaves	Cake	Replacement of wheat flour with florets flour (0, 1.5, 3 and 4.5%) or leaves powder (0, 1, 2 and 3%)	Increased protein, lipids, ash, DF content, TPC, and antioxidant activity	[177]
			Visible effects on color, reducing L* and a*	
			Lower microbial count with increasing level of substitution	
			Decreased energy value and carbohydrate content	
Leaves	GF bread	5% substitution of the original corn starch with broccoli leaf powder (freeze-dried)	Increased protein and mineral content	[178]
			Improved specific volume and bake loss	
			Enhancement of the TPC	
			Improved inhibiting activity against AGEs	
Leaves and stalks	Bread	Replacement of 3 g of wheat flour with broccoli leave or stalk powder	Increased green hue and a higher crust and crumb color intensity	[179]
			Higher TPC and antioxidant capacity	
			Overall acceptance, texture, and appearance were not affected	
Florets	Bread	Replacement of 0, 1, 3, 5, and 7% of wheat flour with broccoli floret powder	Increased DF, protein, and TPC	[180]
			Darker color, reducing lightness by increasing the broccoli powder concentration	
			Harder texture, decreasing viscoelastic parameters	
			Acceptable sensory evaluation at 1 and 3% bread	
Stalks	Biscuits	Substitution of wheat flour with broccoli powder (hot air-drying) at various levels (5, 10, 15%)	Improved texture with increased cohesiveness and reduced brittleness	[182]
			Enhancement of the sensory attributes and overall acceptability	
			Enhanced nutritional quality in terms of the glucosinolate, carotenoid, and phenolic content.	
Florets and stalks	Biscuits	Replacement of 10% of wheat flour with broccoli flour (freeze-dried)	Increased levels of GSLs, carotenoids, and TPC	[183]
			Obtaining a more cohesive and easier to work dough	
			Protection of BACs from thermal degradation by the food matrix	
Stalks	Crackers	12.5 and 15% substitution of the original wheat flour with broccoli stalk powder (freeze-dried)	Increased DF	[185]
			Enhancement of the GSLs content, TPC, and antioxidant capacity	
			Maintenance of the overall acceptance	
			Higher green color and color intensity	
Leaves	Durum wheat pasta	Addition of 2.5 and 5% of broccoli leaf powder (freeze-dried) to the pasta formulation	Improvement of the content of FAAs, FAs, minerals, and dimethyl sulfide	[184,186]
			Decreased optimal cooking time and water absorption	
			Greener color without compromising overall acceptance	
			Firmness and total shearing force decreased	
Sprouts	Durum wheat pasta	Replacement of durum wheat flour with 0, 5, 10, or 15 g/100 g of broccoli sprouts powder (freeze-dried)	Enhancement of the content of protein, lipids, and minerals	[187]
			Higher levels of GSLs, synaptic acid derivatives, TPC, and antioxidant activity	
			Visible effects on color, reducing L* and b*, and increasing a*	
			Increased bitterness in flavor, without affecting the overall quality of the pasta	

TPC: total polyphenol content; AGEs: advanced glycation end-products.

## 7. Conclusions and Future Trends

Due to the growing concern of society and the relevant global authorities for the preservation of the environment, the food industry has focused on achieving greater efficiency and sustainability in its agri-food systems, with the reduction in food waste being a key objective. In addition, a healthy diet is a major concern for consumers, who are increasingly demanding less-processed products with higher nutritional quality. Considering both factors, broccoli and its by-products generated during industrial processing can be an excellent ingredient for other value-added food applications, in the form of food additives or as functional ingredients, improving at the same time the circular economy. Broccoli florets, stems, leaves, and sprouts can be processed into value-added extracts that are particularly useful in the preparation of functional bakery products, replacing the traditional wheat flour with broccoli flour, thus improving their nutritional profile. The incorporation of broccoli and its by-products can satisfy the two interests mentioned above: the valorization of industrial food waste and the benefits against inflammatory, neurological, or cancerous diseases, among others.

Although the beneficial effects of broccoli have been investigated in numerous in vivo and in vitro studies, there is still a lack of knowledge about its application in more diverse foods, such as bakery products. In addition, there are few studies evaluating the stability of certain broccoli compounds as well as the most efficient organic extraction techniques. Further research is needed to obtain more efficient and resistant broccoli extracts that can also be incorporated into new foods without altering their organoleptic characteristics due to their characteristic flavor and color.

In conclusion, *B. oleracea* is a food of great interest for value-added purposes due to its rich content of different BACs beneficial to health. Considering the enormous potential of broccoli, it is essential to take advantage of it and the by-products generated during its processing to carry out an agro-industrial revalorization.

## Figures and Tables

**Table 1:** Proximate composition of diverse botanical parts of broccoli.

Composition	Unit	Florets	Stalks	Leaves	Sprouts	References
Energy	(kcal/100 g FW)	31.00	29.80	50.90	24.13	[1,27,28]
Moisture	(g/100 g FW)	87.00	85.00	90.00	91.06	[29,30]
Ash	(g/100 g DW)	7.62–8.11	10.34–12.37	14.41–18.62	7.38–11.17	[31,32]
Protein	(g/100 g DW)	26.44–31.28	13.73–18.14	21.65–27.49	31.94–43.16	[31,33]
	(g/100 g FW)	2.57	1.00	2.50	2.23–3.00	[1,27,28]
Carbohydrate	(g/100 g DW)	65.13	75.42	66.48	46.70–54.40	[29,33]
	(g/100 g FW)	6.67	4.10	2.30	2.70	[27,28,34]
TSS	(g/100 g DW)	24.61	18.58	25.39	-	[35]
	(mg Glu/g FW)	-	-	-	16.48	[30]
RS	(g/100 g DW)	4.07	2.72	7.07	-	[35]
	(mg Glu/g FW)	-	-	-	4.66	[30]
TDF	(g/100 g DW)	64.42	77.28	62.22	2.10	[35,36]
	(g/100 g FW)	2.40	8.30	10.40	0.36	[1,27,28]
IDF	(g/100 g DW)	58.36	66.18	56.27	1.80	[35,36]
SDF	(g/100 g DW)	6.06	11.10	5.94	0.30	[35,36]
Lipids	(g/100 g DW)	4.59	6.58	6.72	3.16–8.67	[29,33]
	(g/100 g FW)	0.37	0.10	0.50	0.40–0.49	[27,28,34]
SFAs	(%)	28.50	34.30	32.7	12.59–17.72	[29,33]
MUFAs	(%)	9.90	12.70	6.43	14.54–16.05	[29,33]
PUFAs	(%)	62.00	55.10	60.20	68.70–71.61	[29,33]

FW: fresh weight; DW: dry weight; TSS: total soluble sugars; RS: reducing sugars; TDF: total dietary fiber; IDF: insoluble dietary fiber; SDF: soluble dietary fiber; SFAs: saturated fatty acids; MUFAs: monounsaturated fatty acids; PUFAs: polyunsaturated fatty acids.

**Table 5:** Common green extraction techniques and their effects.

Extraction Technique	Basis of the Technique	Outcomes	Ref
UAE	Ultrasound energy to cause fragmentation of the cellular structure	High antioxidant capacity and phenolics and GSLs content	[82]
		Antimicrobial efficiency against *Pseudomonas* spp. and *Candida krusei*	[156]
		Higher myrosinase inactivation and SFN content	[157]
		Antimicrobial activity against *Pseudomonas aeruginosa*	[158]
MAE	Selective heating of polar molecules with microwave energy	Phenolic yield increased up to 65.30, 45.70, and 133.60% for stems, leaves, and florets, respectively	[152]
SFE	Supercritical solvents with low viscosity and high diffusivity facilitate the transport	Antimicrobial efficiency against *Pseudomonas* spp. and *Candida krusei*	[156]
		High yield of β-carotene, PCs, and antioxidant capacity	[13]
		Higher content of non-extractable phenolics and antioxidant capacity	[35]

UAE: ultrasound-assisted extraction; MAE: microwave-assisted extraction; SFE: supercritical fluid extraction.

## Data Availability

No new data were created or analyzed in this study. Data sharing is not applicable to this article.
